# Supplementary material for: Dual roles and evolutionary implications of P26/poxin in antagonizing intracellular cGAS-STING and extracellular melanization immunity
Source: Nat Commun. 2022 Nov 14;13:6934. doi: 10.1038/s41467-022-34761-0 (PMC9663721; doi:10.1038/s41467-022-34761-0)
Supplement: Supplementary file 1 — Supplementary Information [file 41467_2022_34761_MOESM1_ESM.pdf]

Dual roles and evolutionary implications of P26/poxin in antagonizing intracellular cGAS-STING and extracellular melanization immunity

Mengyi Yin[1,2#], Wenhua Kuang[1#], Qianran Wang[1] , Xi Wang[1], Chuanfei Yuan[1], Zhe Lin[3], Huanyu Zhang[1], Fei Deng[1], Haobo Jiang[4], Peng Gong[1*], Zhen Zou[2, 3*], Zhihong Hu[1*], Manli Wang[1*]

[1]State Key Laboratory of Virology, Wuhan Institute of Virology, Chinese Academy of Sciences, Wuhan 430071, China; [2]University of Chinese Academy of Sciences, Beijing 100049, China; [3]State Key Laboratory of Integrated Management of Pest Insects and Rodents, Institute of Zoology, Chinese Academy of Sciences, Beijing 100101, China; [4]Department of Entomology and Plant Pathology, Oklahoma State University, Stillwater, OK, 74078, USA

*To whom correspondence should be addressed:

Manli Wang; Email: wangml@wh.iov.cn

Zhihong Hu; Email: huzh@wh.iov.cn

Zhen Zou; Email: zouzhen@ioz.ac.cn

Peng Gong; Email: gongpeng@wh.iov.cn

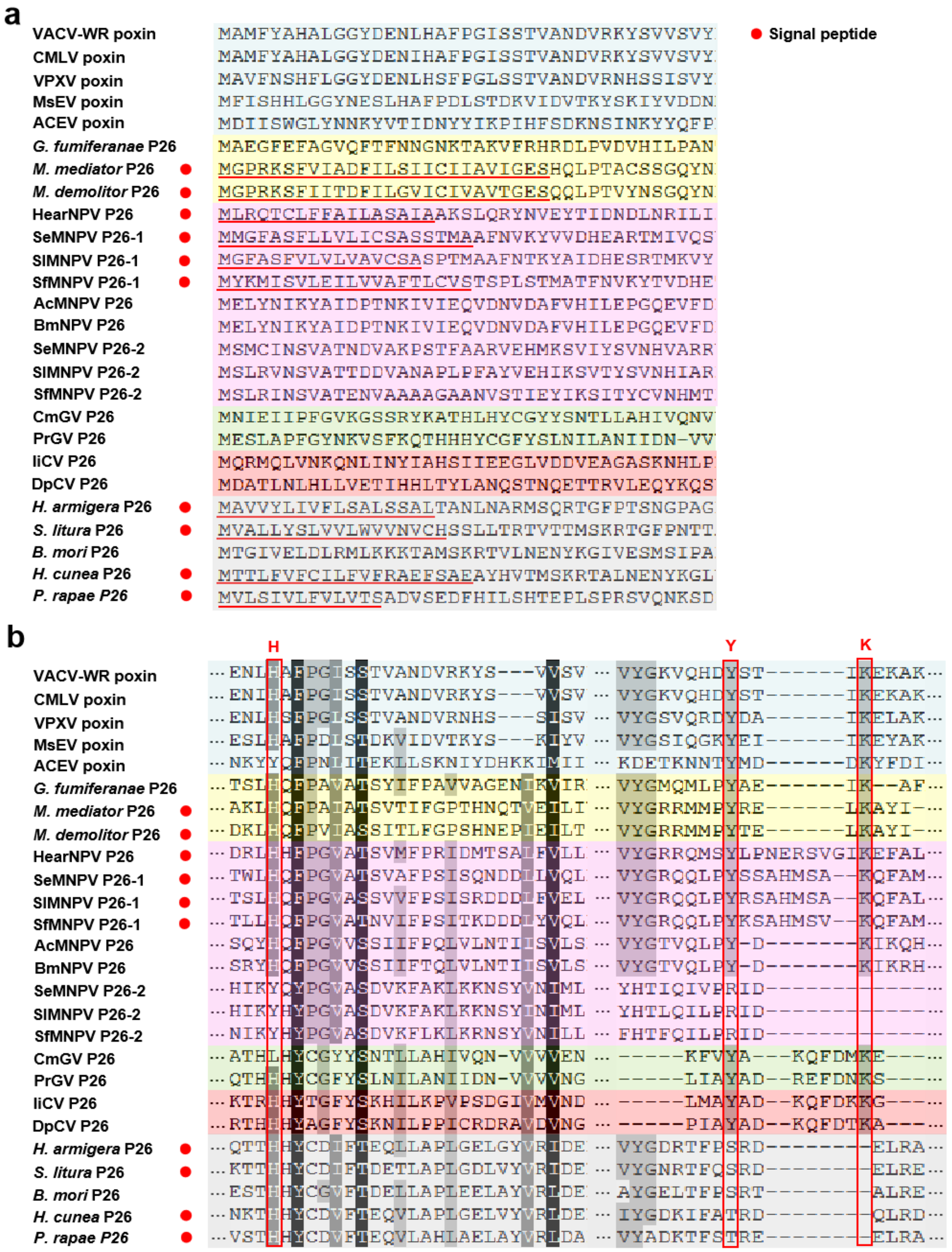

Supplementary Fig. 1 | Analyses of the signal peptide and 2′3′-cGAMP nuclease active site of representative P26/poxin. a, Analysis of signal peptide distribution in representative P26/poxin homologs by the SignalP software. Amino acid sequences were aligned using ClustalW. Signal peptides are underlined in red. **b,** Alignment of conserved amino acids in the representative P26/poxin. The conserved active-site residues of 2′3′-cGAMP catalytic triad are highlighted with red boxes. Sequences

depicted are as follows: VACV (vaccinia virus, accession YP_233066.1 [https://www.ncbi.nlm.nih.gov/protein/YP_233066.1]), CMLV (camelpox virus strain CMS, accession AAG37679.1 [https://www.ncbi.nlm.nih.gov/protein/AAG37679.1]), VPXV (volepox virus, accession YP_009281928.1 [https://www.ncbi.nlm.nih.gov/protein/YP_009281928.1]), MsEV (Melanoplus sanguinipes entomopox virus, NP_048308.1 [https://www.ncbi.nlm.nih.gov/protein/NP_048308.1]), ACEV (Anomala cuprea Entomopoxvirus, accession YP_009001652.1 [https://www.ncbi.nlm.nih.gov/protein/YP_009001652.1]), *G. fumiferanae* (*Glypta fumiferanae*, accession AKD28026.1 [https://www.ncbi.nlm.nih.gov/protein/AKD28026.1]), *M. mediator* (*Microplitis mediator*, accession UXE46284.1, [https://www.ncbi.nlm.nih.gov/protein/ UXE46284.1]), *M. demolitor* (*Microplitis demolitor*, accession NW_014463936.1 [https://www.ncbi.nlm.nih.gov/protein/NW_014463936.1]), HearNPV (Helicoverpa armigera nucleopolyhedrovirus G4, accession NP_075091.1 [https://www.ncbi.nlm.nih.gov/protein/NP_075091.1]), SeMNPV-1 (Spodoptera exigua multiple nucleopolyhedrovirus, accession NP_037889.1 [https://www.ncbi.nlm.nih.gov/protein/NP_037889.1]), SpliMNPV-1 (Spodoptera litura nucleopolyhedrovirus II, accession YP_002332831.1 [https://www.ncbi.nlm.nih.gov/protein/YP_002332831.1]), SfMNPV-1 (Spodoptera frugiperda nucleopolyhedrovirus, accession YP_001036423.1 [https://www.ncbi.nlm.nih.gov/protein/YP_001036423.1]), AcMNPV (Autographa californica multiple nucleopolyhedrovirus, accession NC_001623.1 [https://www.ncbi.nlm.nih.gov/protein/NC_001623.1]), BmNPV (Bombyx mori nucleopolyhedrovirus, accession NP_047534.1 [https://www.ncbi.nlm.nih.gov/protein/NP_047534.1]), SeMNPV-2 (Spodoptera exigua multiple nucleopolyhedrovirus, accession NP_037847.1 [https://www.ncbi.nlm.nih.gov/protein/NP_037847.1]), SplilMNPV-2 (Spodoptera

litura nucleopolyhedrovirus II, accession YP_002332788.1 [https://www.ncbi.nlm.nih.gov/protein/YP_002332788.1]), SfMNPV-2 (Spodoptera frugiperda multiple nucleopolyhedrovirus, accession YP_001036378.1 [https://www.ncbi.nlm.nih.gov/protein/YP_001036378.1]), CmGV (Cnaphalocrocis medinalis granulovirus, accession YP_009229944.1 [https://www.ncbi.nlm.nih.gov/protein/YP_009229944.1]), PrGV (Pieris rapae granulovirus, accession AHD24842.1 [https://www.ncbi.nlm.nih.gov/protein/AHD24842.1]), IiCV (Inachis io cypovirus, accession YP_009002595.1 [https://www.ncbi.nlm.nih.gov/protein/YP_009002595.1]), DpCV (Dendrolimus punctatus cypovirus, accession YP_009111323.1 [https://www.ncbi.nlm.nih.gov/protein/]), *H. armigera* (*Helicoverpa armigera*, accession XP_021193734.1 [https://www.ncbi.nlm.nih.gov/protein/XP_021193734.1]), *S. litura* (*Spodoptera litura*, accession XP_022831420.1 [https://www.ncbi.nlm.nih.gov/protein/XP_022831420.1]), *B. mori* (*Bombyx mori*, accession XP_021205460.1 [https://www.ncbi.nlm.nih.gov/protein/XP_021205460.1]), *H. cunea* (*Hyphantria cunea*, accession AAD09281.1 [https://www.ncbi.nlm.nih.gov/protein/AAD09281.1]), and *P. rapae* (*Pieris rapae*, accession XP_022120734.1 [https://www.ncbi.nlm.nih.gov/protein/XP_022120734.1]).

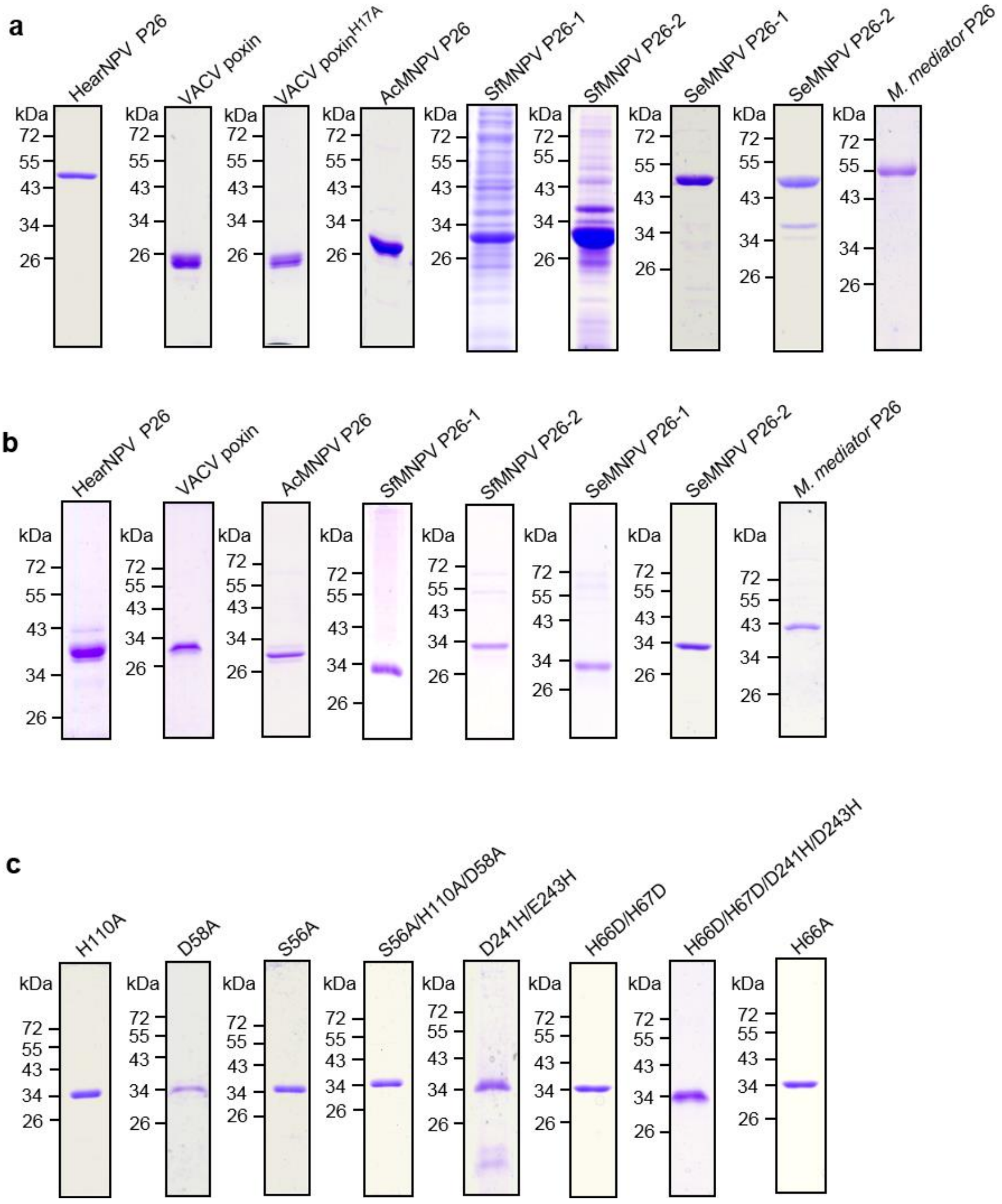

Supplementary Fig. 2 | Sodium dodecyl sulfate polyacrylamide gel electrophoresis (SDS-PAGE) analysis of recombinant P26/poxin proteins. a, Prokaryotic expression of P26/poxin homologous proteins. VACV poxin, VACV poxin^{H17A}, AcMNPV p26, SfMNPV p26-1 and SfMNPV p26-2 were cloned into the **pET-28a (+)** vector and fusion with His tag, HearNPV p26, SeMNPV p26-1, SeMNPV p26-2 and *M. mediaror* p26 were cloned into **pET-32a (+)** vector, fusion with His, Trx and S tag. All proteins

were expressed in the *E. coli* BL21 (DE3) strain and purified by nickel affinity chromatography and a Superdex-200 column. **b,** Eukaryotic expression of P26/poxin homologs. **c,** Eukaryotic expression of HearNPV P26 mutants. All eukaryotic proteins were expressed in a stably transfected *Drosophila* S2 cell line and purified by nickel affinity chromatography and a Superdex-200 column.

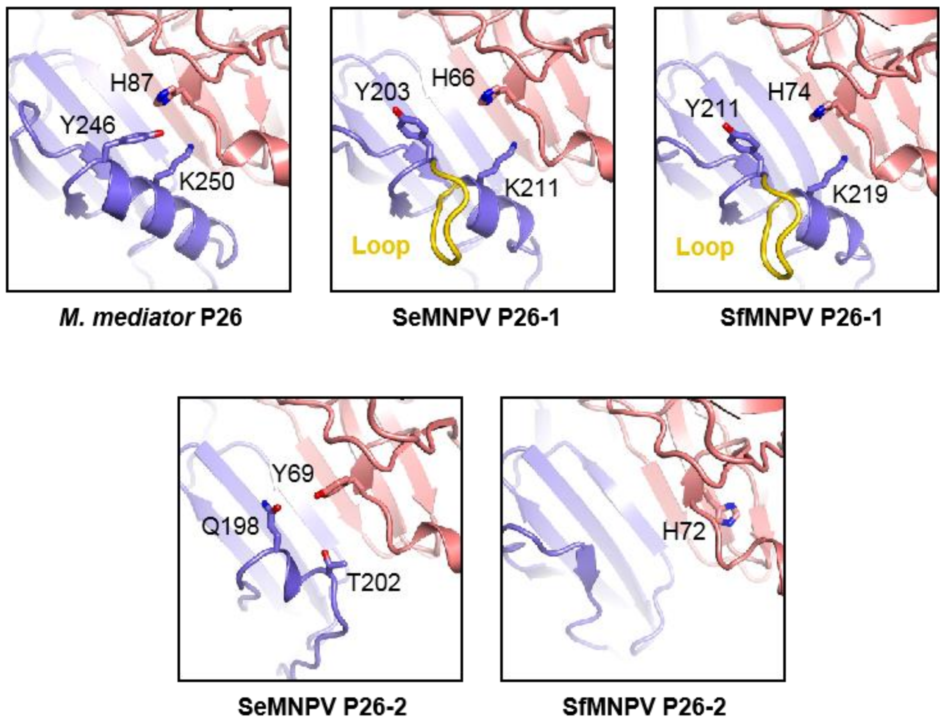

Supplementary Fig. 3 | Comparison of the active site of 2′3′-cGAMP nucleases of HearNPV P26 homologs. The computational structures of *M. mediator* P26, SeMNPV P26-1/P26-2, and SfMNPV P26-1/P26-2 are modeled using the crystal structure of AcMNPV P26 or HearNPV P26 as the templates. The nuclease active-site triad (H-Y-K) shown as sticks is absent in Group II alphabaculovirus P26-2.

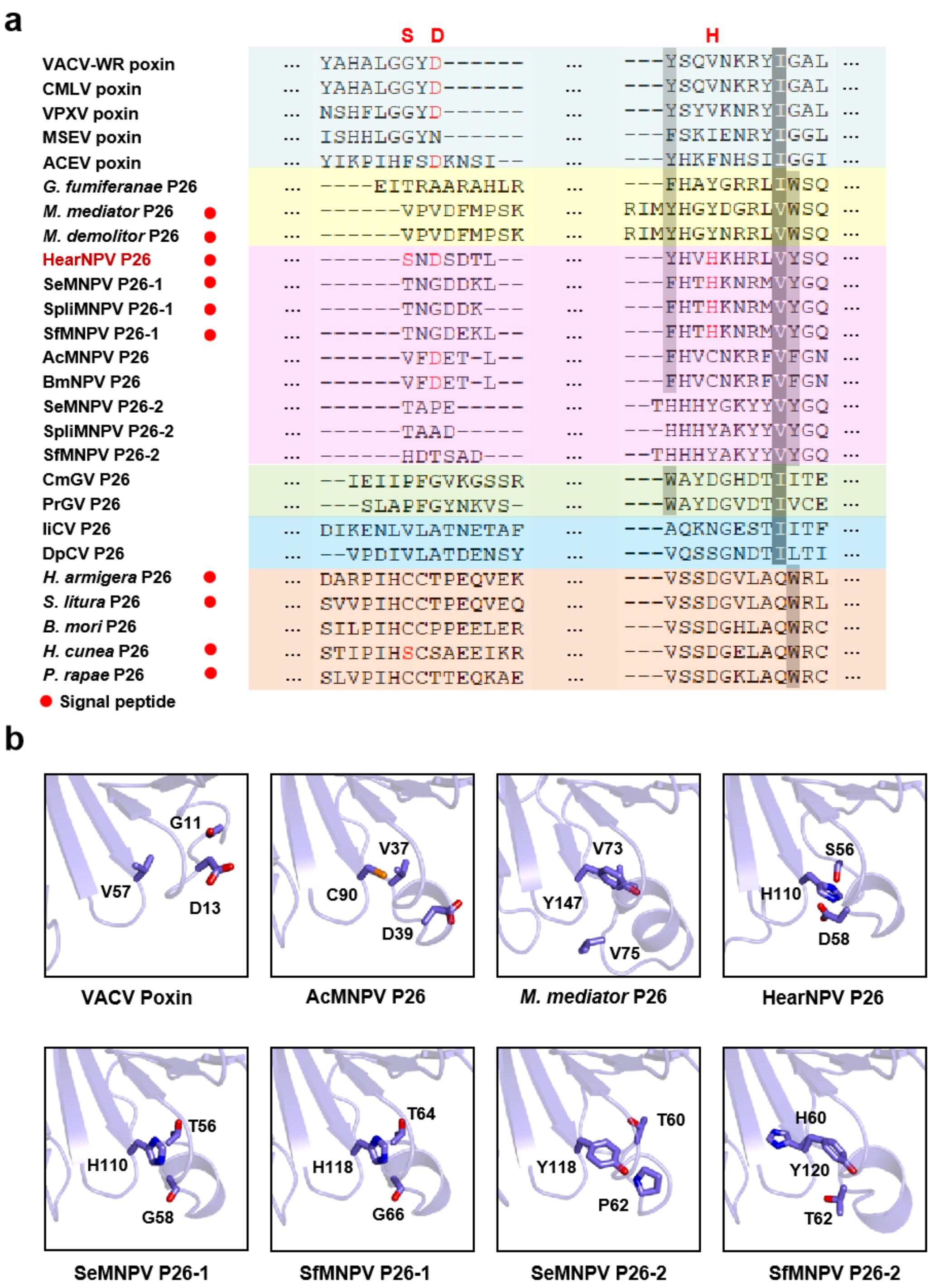

Supplementary Fig. 4 | Evolutionary conservation of a putative serine protease active site in a representative P26/poxin. a, Sequence alignment of poxin and P26 homologs guided by the putative protease catalytic triad (S-H-D). **b,** Structural comparison of the putative protease active site of P26/poxin. The catalytic triad residues are not conserved among P26/poxin homologs.

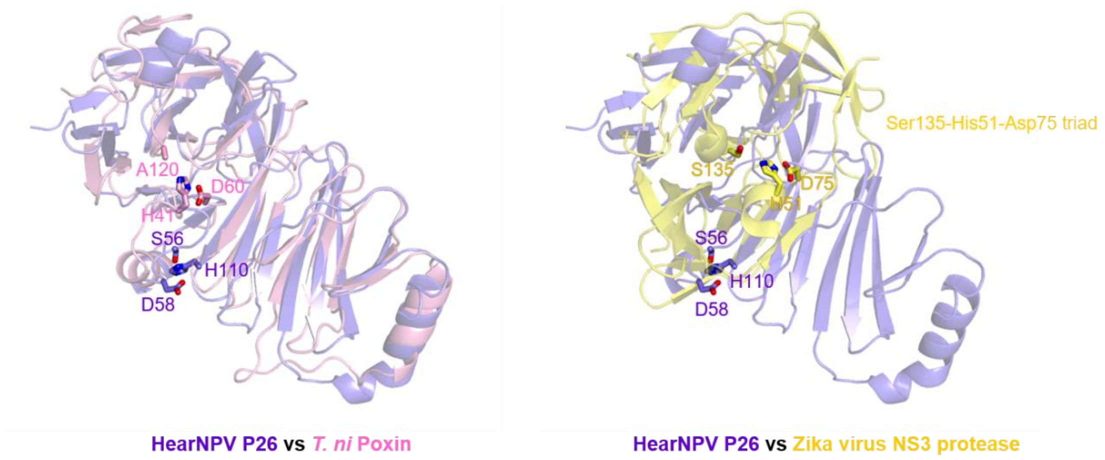

Supplementary Fig. 5 | Structure overlays of HearNPV P26 (PDB:7WN7) versus lepidopteran *Trichoplusia ni* poxin (PDB: 6XB5) and HearNPV P26 versus Zika virus NS3 protease (PDB: 5GPI). The catalytic triad residues of each structure are shown in sticks and highlighted with different colors.

Supplementary Table 1 | X-ray diffraction data collection and structure refinement statistics

Parameter	Value	
	Native P26	SeMet P26
Data collection		
Space group	C2	C2
Cell dimensions		
a, b, c (Å)	194.7, 69.2, 52.7	194.3, 70.4, 52.9
α, β, γ(°)	90.0, 102.1, 90.0	90.0, 102.1, 90.0
Resolution (Å)[a]	30.00-1.90 (1.97-1.90)	30.00-2.70 (2.80-2.70)
No. reflections	52908	19256
R_{merge}	0.072 (0.339)	0.159 (0.331)
R_{meas}	0.081 (0.375)	0.165 (0.344)
$CC_{1/2}$	0.985 (0.952)	0.990 (0.978)
I/sI	15.8 (3.3)	18.6 (9.5)
Completeness (%)	97.1 (94.7)	100 (100)
Redundancy	4.8 (4.6)	13.3 (13.7)
Refinement		
Resolution (Å)	1.90	
No. reflections	51269	
R_{work}/R_{free} (%)[b]	18.3/21.1	
No. atoms		
Protein	3912	
Ligand/ion /water	-/12/ 297	
B-factors (Å^2)		
Protein	31.31	
Ligand/ion/water	-/34.3/36.3	
R.m.s. deviations		
Bond lengths, (Å)	0.007	
Bond angles, (°)	0.918	
Ramachandran statistics[c]	89.4/10.2 /0.5/0.0	
PDB ID code	7WN7	

[a]Values in parentheses are for highest-resolution shell.
[b]5% of data are taken for R_{free} set.
[c]Values are in percentage and are for most favored, additionally allowed, generously allowed, and disallowed regions in Ramachandran plots, respectively.

Supplementary Table 2 | Viral proteins identified in infected *H. armigera* hemolymph

Protein	Test 1				Test 2			
	48 h		72 h		48 h		72 h	
	Number of unique peptides	iBAQ	Number of unique peptides	iBAQ	Number of unique peptides	iBAQ	Number of unique peptides	iBAQ
P26	6	1.65E+08	4	5.07E+07	6	1.47E+08	4	6.25E+07
Cathepsin	2	4.33E+07	2	1.82E+08	1	2.25E+07	2	1.24E+08
Ubiquitin	2	3.80E+07	2	1.04E+08	2	2.81E+07	2	3.70E+07
EGT	3	1.56E+07	4	4.67E+07	4	2.62E+07	4	2.51E+07
SOD	1	9.85E+06	2	7.49E+07	1	4.98E+06	2	3.10E+07
FGF	2	7.83E+06	1	1.62E+06	2	4.56E+06	1	3.01E+06
HA69	1	7.81E+06	1	8.55E+06	0	0.00E+00	1	7.57E+06
HA81	3	4.14E+06	3	1.00E+07	4	4.03E+06	3	8.54E+06
HA26	1	3.17E+06	1	9.80E+06	1	3.29E+06	1	6.22E+06
PEP	1	1.80E+06	2	6.39E+06	1	7.54E+05	2	3.90E+06
HA115	1	1.63E+06	1	1.82E+06	1	1.58E+06	1	1.26E+06
Polyhedrin	1	1.19E+06	2	6.36E+06	2	9.63E+05	2	4.73E+06
P48	3	1.06E+06	2	3.43E+06	2	1.21E+06	2	2.38E+06
F protein	1	5.69E+05	2	1.45E+06	2	1.09E+06	2	9.16E+05
HA66	1	2.35E+05	2	1.72E+06	1	2.37E+05	2	1.69E+06
HA44	1	9.27E+04	1	6.61E+05	1	9.19E+04	1	2.43E+05
FP25K	1	0.00E+00	2	8.94E+06	0	0.00E+00	2	3.88E+06
Chitinase	0	0.00E+00	7	3.25E+06	0	0.00E+00	4	5.77E+05
39K	0	0.00E+00	1	4.21E+05	0	0.00E+00	1	4.38E+05
LEF-6	0	0.00E+00	2	4.27E+06	0	0.00E+00	1	4.15E+05

Supplementary Table 3 | Bioassay results in third-instar *H. armigera* larvae

Virus	Median lethal concentration		Median lethal time		
	LC_{50} (95% CL) (10^4 OBs/mL)	Potency ratio (95% CL)	ST_{50} (95% CL) (h)	χ^2	*P*
Wild-type	2.0 (1.2, 3.5)	—	96 (93.9, 98.1)	—	—
Δ*p26*	2.3 (1.4, 3.7)	0.892 (0.568, 1.400)	102 (98.5, 105.5)	9.137	0.003
RE*p26*	3.8 (2.3, 6.3)	0.541 (0.314, 0.932)	96 (94.4, 97.6)	0.131	0.718

LC_{50} values were calculated by probit regression analysis, and further compared using two-sided potency ratio test. The ST_{50} values were calculated using the Kaplan-Meier estimator and further compared using two-sided Log-rank test.
