# Supplementary material for: Dual roles and evolutionary implications of P26/poxin in antagonizing intracellular cGAS-STING and extracellular melanization immunity
Source: Nat Commun. 2022 Nov 14;13:6934. doi: 10.1038/s41467-022-34761-0 (PMC9663721; doi:10.1038/s41467-022-34761-0)
Supplement: Supplementary file 3 — Reporting Summary [file 41467_2022_34761_MOESM3_ESM.pdf]

nature portfolio

Corresponding author(s): Manli Wang, Zhihong Hu, Zhen Zou, Peng Gong

Last updated by author(s): Nov 3, 2022

Reporting Summary

Nature Portfolio wishes to improve the reproducibility of the work that we publish. This form provides structure for consistency and transparency in reporting. For further information on Nature Portfolio policies, see our Editorial Policies and the Editorial Policy Checklist.

Statistics

For all statistical analyses, confirm that the following items are present in the figure legend, table legend, main text, or Methods section.

n/a	Confirmed	
☐	☒	The exact sample size (*n*) for each experimental group/condition, given as a discrete number and unit of measurement
☐	☒	A statement on whether measurements were taken from distinct samples or whether the same sample was measured repeatedly
☐	☒	The statistical test(s) used AND whether they are one- or two-sided<br>*Only common tests should be described solely by name; describe more complex techniques in the Methods section.*
☒	☐	A description of all covariates tested
☒	☐	A description of any assumptions or corrections, such as tests of normality and adjustment for multiple comparisons
☐	☒	A full description of the statistical parameters including central tendency (e.g. means) or other basic estimates (e.g. regression coefficient) AND variation (e.g. standard deviation) or associated estimates of uncertainty (e.g. confidence intervals)
☐	☒	For null hypothesis testing, the test statistic (e.g. *F*, *t*, *r*) with confidence intervals, effect sizes, degrees of freedom and *P* value noted<br>*Give P values as exact values whenever suitable.*
☒	☐	For Bayesian analysis, information on the choice of priors and Markov chain Monte Carlo settings
☒	☐	For hierarchical and complex designs, identification of the appropriate level for tests and full reporting of outcomes
☒	☐	Estimates of effect sizes (e.g. Cohen's *d*, Pearson's *r*), indicating how they were calculated

Our web collection on statistics for biologists contains articles on many of the points above.

Software and code

Policy information about availability of computer code

Data collection	Gen5, Gel Capture MicroChemi v2.2.2.0, CFX Manager 3.0, HKL2000 v708, Q Exactive HF coupled with an UltiMate 3000 RSLCnano system.
Data analysis	GraphPad Prism v8.0.2, SPSS Statistics v22, Mascot v2.3, PHENIX Suite v1.11.1-2575 and the Phaser program is included in the PHENIX software package, Coot v0.8.9.2, THESEUS v3.3.0, MaxQuant V1.6.6 software.

For manuscripts utilizing custom algorithms or software that are central to the research but not yet described in published literature, software must be made available to editors and reviewers. We strongly encourage code deposition in a community repository (e.g. GitHub). See the Nature Portfolio guidelines for submitting code & software for further information.

Data

Policy information about availability of data

All manuscripts must include a data availability statement. This statement should provide the following information, where applicable:

- Accession codes, unique identifiers, or web links for publicly available datasets
- A description of any restrictions on data availability
- For clinical datasets or third party data, please ensure that the statement adheres to our policy

The data generated in this study are provided in the Source Data and also available from the corresponding authors upon request. Atomic coordinates and structure factors for the crystal structure generated in this study have been deposited in the Protein Data bank (https://www.rcsb.org) under PDB code 7WN7 (https://www.rcsb.org/structure/unreleased/7WN7). Previously published crystal structures used in this study are available from the PDB codes 6EA9 (https://

doi.org/10.2210/pdb6EA9/pdb), 6XB3 (https://doi.org/10.2210/pdb6XB3/pdb), 6XB5 (https://doi.org/10.2210/pdb6XB5/pdb) and 5GPI (https://doi.org/10.2210/pdb5GPI/pdb). The mass spectrometry proteomics data have been deposited in the ProteomeXchange Consortium with the dataset identifier PXD037784 (http://proteomecentral.proteomexchange.org/cgi/GetDataset?ID=PXD037784). Source data are provided with this paper.

Human research participants

Policy information about studies involving human research participants and Sex and Gender in Research.

Reporting on sex and gender	N/A
Population characteristics	N/A
Recruitment	N/A
Ethics oversight	N/A

Note that full information on the approval of the study protocol must also be provided in the manuscript.

Field-specific reporting

Please select the one below that is the best fit for your research. If you are not sure, read the appropriate sections before making your selection.

☒ Life sciences ☐ Behavioural & social sciences ☐ Ecological, evolutionary & environmental sciences

For a reference copy of the document with all sections, see nature.com/documents/nr-reporting-summary-flat.pdf

Life sciences study design

All studies must disclose on these points even when the disclosure is negative.

Sample size	For PO activity assays of individual insect, we tested insect sample size of n= 5,10,15,25,50 and 100, respectively. The results showed no significant difference via two-tailed Student's t-test.<br>For qPCR and LC-MS/MS assays, the sample size was chosen according to previous study (Yuan et al. et al., 2017 PMID:28953952).<br>For oral infectivity assays of the recombinant viruses, we followed the sample size used in previously study [Sun, X. et al. Biological activity and field efficacy of a genetically modified Helicoverpa armigera single-nucleocapsid nucleopolyhedrovirus expressing an insect-selective toxin from a chimeric promoter. Biol. Control 29, 124–137 (2004)].
Data exclusions	No data were excluded from analyses.
Replication	For PO activity assays of individual insect, we infected 5 larvae at one time and we permormed infection three times. For qPCR assays of individual insect, we infected 5 larvae at one time and we permormed infection twice. Infection were performed independently with the interval approximately 1 month according to the life cycle of insects.<br>For inhibitory effect of PO activation and LC-MS/MS, hemolymph was a mixture from 100 to 200 healthy larvae for every replicate. Hemolymph collection were performed independently with the interval approximately 1 month according to the life cycle of insects.<br>For inhibitory effect of PO activation and 2′3′-cGAMP nuclease activity assays used proteins purified 3 times for 3 replicates.<br>Attempts of data replication were successful.
Randomization	Insects were randomized into control and experimental groups.<br>To analyze the inhibitory effect of PO activation, in one time of experiment, hemolymph was a mixture from 100 to 200 healthy larvae selected randomly.
Blinding	Blinding was not performed for data analysis or group allocation, as insects were randomly assigned to each experimental group. Data were collected by unbiased quantitative means, as there was no subjective measurement in our experiments.

Reporting for specific materials, systems and methods

We require information from authors about some types of materials, experimental systems and methods used in many studies. Here, indicate whether each material, system or method listed is relevant to your study. If you are not sure if a list item applies to your research, read the appropriate section before selecting a response.

Materials & experimental systems

n/a	Involved in the study
	☒ Antibodies
	☒ Eukaryotic cell lines
☒	Palaeontology and archaeology
	☒ Animals and other organisms
☒	Clinical data
☒	Dual use research of concern

Methods

n/a	Involved in the study
☒	ChIP-seq
☒	Flow cytometry
☒	MRI-based neuroimaging

Antibodies

Antibodies used

Abcam anti-V5 tag antibody (ab15828);
Affinity anti-rabbit IgG HRP (S0001).
Anti-HearNPV P26, procSPH11, procSPH50 and HSP27.2 were generated in our laboratory. The prokaryotic expressed of recombinant proteins HearNPV P26, procSPH11, procSPH50 and HSP27.2 were used for rabbit immunization to elicit the production of polyclonal antibody.

Validation

Abcam anti-V5 tag antibody, as noted on the manufacturer's webpage, this antibody has previously been validated and cited by over 40 publications (Kennedy A.L. et al. et al., 2021 PMID:33637765 and Boulay G. et al., 2021 PMID: 33361335).
Anti-procSPH11 and procSPH50 antibodies were validated for use in Helicoverpa armigera (Wang et al. et al., 2020 PMID:32431706).
Anti-HSP27.2 antibody was validated for use in Helicoverpa armigera (Yuan et al. et al., 2017 PMID:28953952).
Anti-HearNPV P26 antibody was generated from rabbit immunization. It specificity has been validated by our laboratory.

Eukaryotic cell lines

Policy information about cell lines and Sex and Gender in Research

Cell line source(s)

The S2 cells (Invitrogen, R69007) were a gift from Prof. Tianwei Lin from Xiamen University. The HzAm1 cells were were a gift from Prof. Just Vlak of Wageningen University.

Authentication

The S2 cells and HzAm1 cells were not authenticated.

Mycoplasma contamination

Cell line was not tested for mycoplasma contamination.

Commonly misidentified lines
(See ICLAC register)

No misidentified lines were used.

Animals and other research organisms

Policy information about studies involving animals; ARRIVE guidelines recommended for reporting animal research, and Sex and Gender in Research

Laboratory animals

Helicoverpa armigera, male and female, third instar larvae.
Spodoptera frugiperda, male and female, fourth instar larvae.
Rabbit, male, about 2 kg.

Wild animals

This study did not involve wild animals.

Reporting on sex

N/A

Field-collected samples

This study did not involve field-collected samples.

Ethics oversight

Ethics statement for rabbit immunization followed the Institutional Animal Care and Use Committee (IACUC) of the Wuhan Institute of Virology, Chinese Academy of Sciences (ethics number: WIVA01201601).

Note that full information on the approval of the study protocol must also be provided in the manuscript.
